# An Experimental Study on the Influence of Friends and Popular Peers on Adolescents’ Risky Decisions in a Private and Public Context

**DOI:** 10.1007/s10964-026-02349-2

**Published:** 2026-04-15

**Authors:** Scarlett K. Slagter, Ana da Silva Pinho, Anna C.K. van Duijvenvoorde, Wouter van den Bos

**Affiliations:** 1 Department of Psychology, University of Amsterdam, 1018 WS Amsterdam, The Netherlands; 2 Department of Developmental and Educational Psychology, Institute of Psychology, Leiden University, 2333 AK Leiden, Netherlands; 3 Leiden Institute for Brain and Cognition, 2333 AK Leiden, Netherlands; 4 Amsterdam Brain and Cognition, University of Amsterdam, 1018 WS Amsterdam, The Netherlands; 5 Max-Planck-Institut für Bildungsforschung, 14195 Berlin, Germany

**Keywords:** Peer Influence, Friends, Popular Peers, Risky Decision-Making, Adolescence, Choice Disclosure

## Abstract

Understanding why peers influence adolescents’ risky decisions requires identifying their primary sources of influence. This study compared the influence of friends and popular peers on adolescents’ risky decision-making within a high school setting. The sample consisted of 457 Dutch adolescents, aged 11–18 years (mean age = 14.32, SD = 1.86), with 53% identifying as female. Adolescents and their potential influencers were recruited from high schools. At these high schools, adolescents played the Columbia Card Task (CCT), during which they observed the choices made by their friends or popular classmates (within-condition). Participants were randomly assigned to either a private or public condition of the task (between-condition). Their final decisions were either disclosed to these peers (public) or kept confidential (private). This study showed that friends and popular peers both influence adolescents’ choices, with only slightly greater adjustments for friends. This was found for both contexts (private or public). Adolescents adjusted their initial choices in 88% of cases, with adjustment strategies largely depending on whether peers’ choices were more or less risky; Riskier choices were more often followed or even exceeded, while more cautious choices were more frequently ignored. As a result, peers had a stronger effect on increasing risk-taking than reducing risk-taking. These results inform a discussion on adolescents’ motivational drivers of adjustment and the implications of heightened sensitivity to displaying risky behaviour in peer contexts.

**Supplementary Information:**

The online version contains supplementary material available at 10.1007/s10964-026-02349-2.

## Introduction

The decision to take a risk is not straightforward and often uncertain. Individuals must weigh the potential consequences of their actions, but they often lack complete information about the possible outcomes. For example, when deciding to drive a scooter without a helmet, one must consider the likelihood and/or magnitude of possible injuries against the potential benefits. Given the inherent uncertainty of risky decisions, people might turn to others for information. In particular, adolescents’ tendency to take or avoid risks seems dependent on social signals from their peers; cautious peers can stimulate more safe choices, while peers with a riskier attitude can sway adolescents to make more risky choices (Braams et al., 2019; Cascio et al., 2015; Knoll, Leung, Foulkes & Blakemore, 2017). However, not all peers exert the same influence due to their social relationships; some may have higher social status, such as popular peers, while others may share more similarities, like best friends. In addition to the ambiguity in risk-decisions that stimulates adolescents to seek guidance from peers (Slagter et al., 2023a ), many risk-behaviours occur when peers are present. In these situations, risk-taking might not always be rational, driven by one’s risk assessment, but rather guided by what is socially accepted or what benefits one’s social status. To understand how adolescents’ risk behavior is influenced by their peers, it is essential to identify and understand which peers serve as a source of influence in a private and public context. This understanding is especially important for behavior change interventions that focus on adolescents’ social networks (Paluck et al., 2016; Veenstra & Laninga-Wijnen, 2022). This study therefore examined how two different peer sources within adolescents’ school-based social networks influence adolescents’ risky choices. Specifically, it investigated these influences in contexts where adolescents’ choices were either visible or not visible to their peers.

### The Role of Friends and Popular Peers in Adolescent Risk-taking

Previous literature has identified two major sources that impact adolescent risk-taking: friends and popular peers. For example, adolescents’ perceptions of their popular peers’ substance use at high school predicted steeper increases in adolescents’ own substance use over a 2-year period (Helms et al., 2014), and exposure to anti-alcohol messages from peers with a high social status was shown to reduce adolescents’ willingness to drink (Teunissen et al., 2012). However, the impact of popular peers is mixed and not consistently observed (Field et al., 2023; Field & Prinstein, 2023, but also see: Slagter, Gradassi, van Duijvenvoorde et al., 2023; Lansu et al., 2015). Furthermore, the impact of popular peers has been primarily established in comparisons to low-status peers only (e.g. Cohen & Prinstein, 2006; Teunissen et al., 2012). Remarkably, only a few studies have simultaneously examined the relative impact of friends and popular peers. For example, a longitudinal study showed that the injunctive norm of popular peers (i.e., adolescents’ perceptions of their peers’ attitudes regarding substance use) did not predict adolescents’ onset of substance use over time beyond the injunctive norm of friends (Field, 2023). In addition, an information sampling study showed that adolescents were mainly interested in receiving information from their friends but not from peers perceived as popular (Slagter et al., 2023b). Thus, these studies suggest that the impact of friends may be larger than that of popular peers. As adolescents are exposed to both friends and popular peers in their daily lives, it will be important to directly assess the relative influence of friends and popular peers within the same study.

### Motivational Factors for Conformity in a Private and Public Context

The influence of friends and popular peers may vary depending on whether decisions are made in private settings, such as scrolling through Instagram at home, where choices remain confidential, or in public contexts, like being in the school canteen surrounded by classmates, where peers can observe and evaluate one’s choices. In these different contexts, different motives for behavioral conformity may be at play (Deutsch & Gerard, 1955; Cialdini & Goldstein, 2004).

In private contexts where one’s actions or decisions remain confidential, two motives may explain adolescents’ tendency to adjust their behavior. In novel and uncertain situations, peer choices can offer valuable guidance on what actions to take. Indeed, adolescents are particularly inclined to observe others (Slagter, et al., 2023a) and rely on their peers’ choices when they feel uncertain about their own preferences (Reiter et al., 2021). In such cases, the choices of similar peers, like friends (Laursen, 2017), can be seen as more informative for shaping one’s preferences than those of non-similar peers (Analytis et al., 2018). Additionally, adolescents may model and copy others’ behavior in private settings due to their need to belong and affiliate with peers (Baumeister & Leary, 1995). Social identity theory (Tajfel & Turner, 1986) states that individuals are inclined to match the behavior of groups with a shared identity to foster a sense of belonging and to maintain a positive social identity with this group. During adolescence, friends become one of the most important social identities. As experiencing dissimilarity with their friends’ behavior would disrupt one’s sense of belonging with this group, adolescents will be motivated to align with the behavior of their friends to reinforce their feeling of shared identity (Laursen, 2017; Laursen & Veenstra, 2021).

Risky decisions are also often made in public settings. In these contexts, peers first observe the wishes or actions of others and decide on their actions while these peers are still around. This allows other peers to evaluate one’s decisions and actions. In such a setting, adolescents might also adjust their behavior to prevent peer rejection and to foster positive social evaluation from their peers (Somerville, 2013; Westenberg et al., 2007). Matching the behavior of others could foster peer acceptance and, while being behaving differently could lead to peer rejection (Blakemore, 2018; Falk et al., 2014). This motive is often cited as an explanation for why adolescents exhibit more risky behavior in the presence of peers compared to being alone (e.g., Falk et al., 2014; Chein et al., 2011;Gardner & Steinberg, 2005). Adolescents may particularly fear social rejection by popular peers, as these classmates can damage their social status in the class. Popular peers often hold a central position in class and are seen as powerful due their dominant and intimidating behavior or because they are well-liked and admired (Cheng et al., 2013; De Bruyn & Cillessen, 2006b; van den Berg et al., 2015). Peer acceptance from these popular peers can enhance one’s social status within the peer group (Dijkstra et al., 2010). By contrasting peer influences in private and public context, it is possible to examine whether the influence of popular peers mainly relates to the need for positive peer evaluation (public setting), whereas the influence of friends is already present in private contexts due to preference uncertainty or the need to maintain a strong sense of belonging with this in-group.

### Age Differences in Peer Influence Sources

Age-effects on the influence of peers in general have been extensively studied, but not the influence of specific peers. Previous literature suggests that the importance of being popular and having intimate friendships might change with age. When adolescents get older, the importance of social status might be overruled by their need to maintain their good friendships, and therefore the susceptibility for popular peers might decrease with age (Gavin & Furman, 1989; Lafontana & Cillessen, 2010). Only a few empirical studies have examined whether the strength of specific sources of peer influence varies across adolescence, yielding mixed results. Pinho et al. (2022) found no age effect on adolescents’ susceptibility to popular peers from ages 11 to 18. In contrast, other research suggests that adolescents’ sensitivity to popular peers is absent in early adolescence but emerges in mid-adolescence (Field et al., 2023; Cohen & Prinstein, 2006). A recent study also showed that adolescents prefer to learn about their friends’ risk preferences over those of non-friends, and this bias for friends increased with age (Slagter et al., 2023b). Together, these mixed and limited findings highlight the importance of mapping developmental changes in susceptibility to various sources of peer influence, particularly friends versus popular peers.

## Current Study

Previous research has shown that friends and popular peers can be key sources of influence, but the relative influence of these sources remains unclear. This preregistered study aimed to examine the influence of friends and popular peers from school on adolescents’ risky decision making. Participants (*N* = 457, aged 11–18 years) played the Columbia Card Task (CCT) alone, risking points by turning over cards. In a follow-up session, they also observed choices made by either a friend or a popular peer before making their own decisions, with social source manipulated within subjects. This was examined across two decision contexts to assess whether the relative influence of social sources was context dependent. In the private context, choices remained confidential, whereas in the public context, choices were disclosed to familiar peers (between-subjects). As preregistered, it was hypothesized that friends would have a stronger influence in the private context compared to popular peers (Hypothesis 1). In addition, it was hypothesized that the influence of popular peers would be context dependent, leading to a greater influence when choices are disclosed to popular peers compared to when they are kept private (Hypothesis 2). Consequently, in the public context, the relative difference in influence between friends and popular peers would be smaller. Exploratively, age variations in adolescents’ sensitivity to friends and popular peers were assessed.

## Methods

### Participants

Adolescents and their familiar peers were recruited by targeting high-school classes from 6 high schools in the Netherlands. The analyzed sample consisted of 457 participants aged 11–18 years (53% identified as female, Mage = 14.32, SD = 1.86) from 35 classrooms. A total of 226 participants were assigned to the private condition, and 231 participants were assigned to the observation condition. Table 1 shows the demographic details for each context condition. Informed consent was obtained from all participants prior to the study. For minors, also written informed consent was given by their parents or legal guardians. Recruiting adolescents from high school classes allowed us to include participants and their friends and popular peers from class as potential influencers in the study. Thus, the data of participating students (by some seen as friend or poplar) could potentially also be used to design the social conditions of the experimental task. All students were informed about this.

**Table 1:** Demographic characteristics of the participants

	Observation context	Private context	Full Sample
	*N* (%)	*N* (%)	*N* (%)
Gender Female Male	124 (54) 107 (46)	118 (53) 108 (47)	242 (53) 215 (47)
Age range median	11–18 14	11–19 15	11–19 14

#### Suitability of Targeting Adolescents’ Social Network at School

In the Dutch school system, adolescents transition to high school at around 11 years old. Upon entering high school, students are placed into fixed groups of classmates and follow educational lessons and activities together. These groups remain the same throughout their program, which lasts 5 or 6 years depending on the educational track. Data-collection took place in the second half of the year to make sure first year’s students would know their classmates for several months.

#### Recruitment Strategy and Participation Rate per Classroom

Several secondary schools representing different educational tracks (pre-vocational, general, and pre-university education) were approached to participate in the study. Where possible, multiple classes from different grade levels within a school were included. Schools selected classes whose mentors considered participation feasible during scheduled mentor hours. All students in these classrooms were invited to participate. Students without parental or own consent were instructed to do homework in class. Schools received monetary compensation of €5 per participant for each session. This amount had to be spent on a school outing chosen by the students. Students were aware of this compensation and their say in the decision. The participation rate per class ranged from 64% to 100%, with a mean rate of 85% (SD = 12.47; see Supplemental Materials Table S6 for a list of participation rates). During data collection, the recruitment strategy obtained a focus on classes from specific grade levels to achieve a balanced age distribution. Recruitment ended once the target sample size was reached, accounting for potential participant dropouts.

#### Rationale Sample Size and excluded Participants

Based on the estimated effect sizes from a pilot study (*N* = 32), it was predetermined that a sample of *N* = 200 would provide 85% power to detect a moderate interaction effect between social source (within-level) and decision context (between-level). In total, 36 classes from 6 high schools in the Netherlands participated in this study resulting in a total sample size of 590 participants aged 11–19 years. Participants for whom the social source conditions or the observation condition of the task could not be constructed were excluded from the data analysis (e.g., choices from popular peers were unavailable because they did not participate in the study; *N* = 114). The sample size was further reduced during data processing and cleaning due to our preregistered exclusion criteria (*N* = 19). See the Supplemental Materials Table S2 for more details on the exclusion criteria and the exclusions. These exclusions resulted in a final sample of 457 participants aged 11–18 years (53% identified as female, Mage = 14.32, SD = 1.86) from 35 classrooms. For this sample, no data points were missing for the planned analysis.

### Procedure

**Fig. 1:**
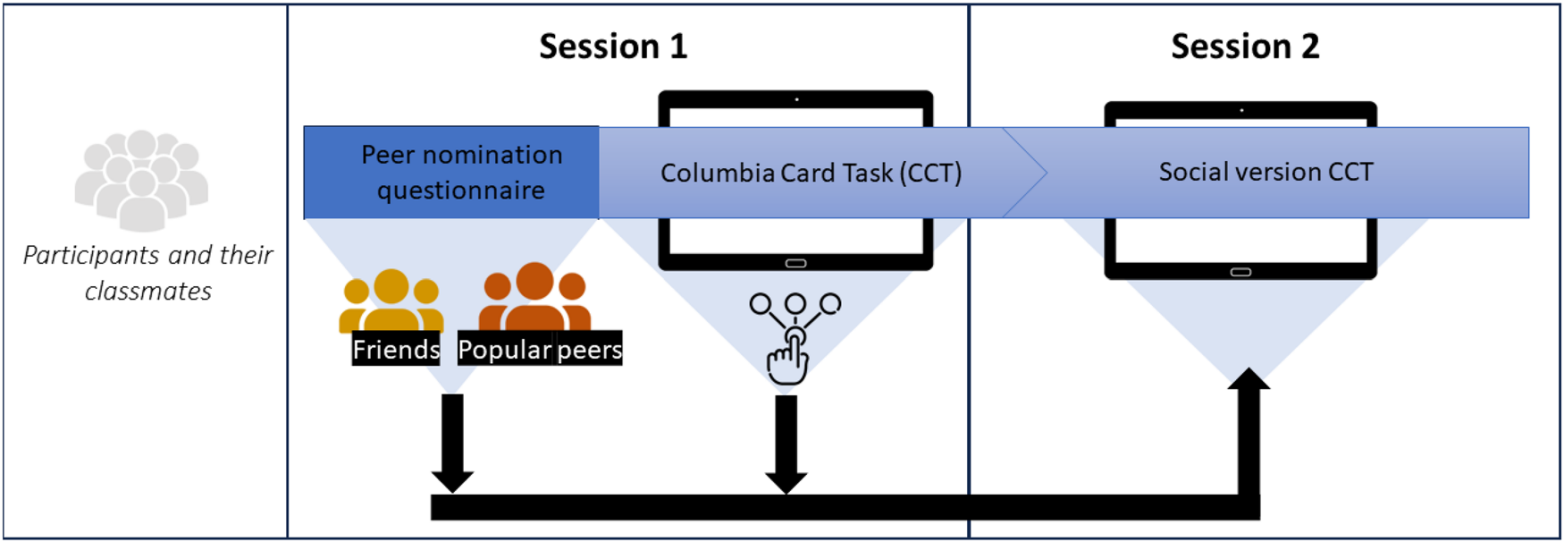
Flowchart of the experimental design tailored at the individual level. Participants and their classmates participated in two sessions. In the first session, participants indicated which classmates were their friends and which classmates were most popular in class. These nominations were used to construct the two social sources within the social version of the Columbia Card Task (CCT), a risky decision-making task. Participants and their classmates performed this task alone during session 1 to record all individual choices. In session 2, participants performed the CCT again but observed the choice made by a nominated friend or nominated popular peer.

The study took place at schools in classrooms, where screens were divided into tables to ensure privacy for all pupils when performing the experiment. The data were collected in 2 sessions with two- or three-week intervals (see Fig. 1).

In the first session, participants and their classmates completed a peer nomination questionnaire to identify their (best) friends and the classmates they perceived as most popular. Afterwards, they played the Columbia Card Task (CCT), during which their initial risky choices were recorded over 20 rounds.

In the second session, participants played the CCT again, but this time they were exposed to real-made choices from two types of peers to assess how their decisions would be influenced by the behavior of these specific sources. Participants viewed choices made by both a friend and a popular peer in a within-subjects design, with 10 decision rounds for each type of peer. The order of presenting choices from the friend or popular peer group was randomized across participants. Participants were assigned to either the private or observation condition of the task (between-subjects; see Supplemental Materials ‘Allocation procedure’ for details). In the private condition, participants’ choices were kept privately. In the observation condition, participants were informed that their final choices would be shared with either their friend or a popular classmate. Participants were unaware of the different decision contexts (private vs. peer observation) of the study.

Each session lasted approximately 45 min. To incentivize the performance of the CCT, points collected in the CCT were converted into lottery tickets for a €40 online shopping voucher. All procedures were approved by the Ethics Review Board Faculty of the University of Amsterdam (2021-COP-14082) and performed in accordance with relevant guidelines and regulations.

### Experimental Design

#### Solo Choices in the Columbia Card Task

**Fig. 2:**
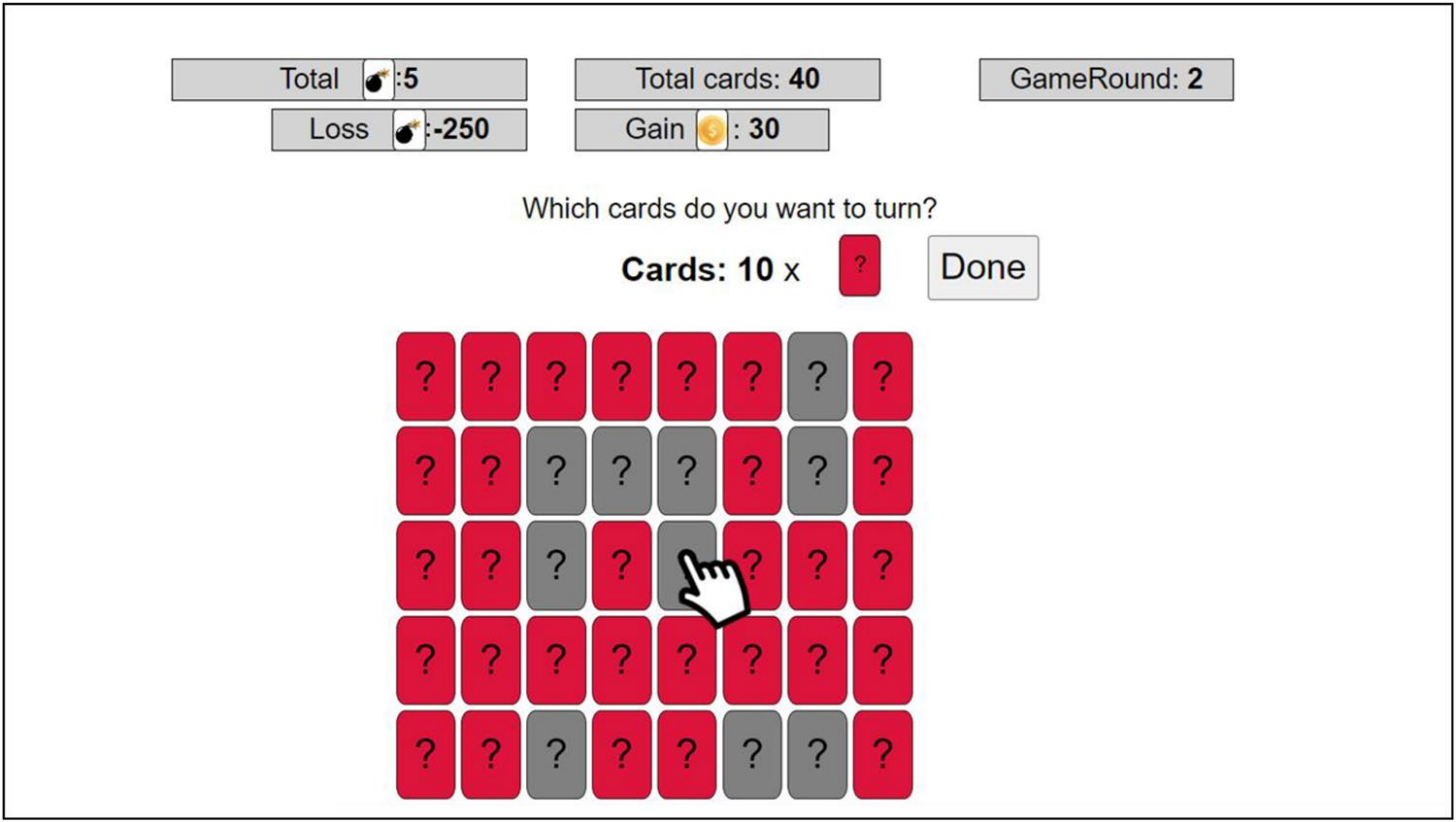
Decision screen from the Columbia Card Task.The Columbia Card Task (CCT) is a risky card-turning game in which participants earn points by turning over gain cards and lose points if they turn over bomb cards. The total number of cards, the odds of losing and the gain amount vary trial-by-trial and are displayed at the top of the screen. In this solo version of the CCT, participants decided on how many cards they want to turn over by selecting these cards. Here, an increased number of cards selected was seen as greater risk-taking.

Adolescents’ risky decision making was measured with the Columbia Card Task (CCT), which involves mathematical calculations and deliberate reasoning (Figner et al., 2009). In this task, participants decided how many face-down cards they wanted to turn over from a set of 32 or 40 cards, which included both gain and bomb cards (see Fig. 2). Selecting a gain card resulted in gaining 30 or 40 points, depending on the decision round. Selecting a bomb card resulted in a loss of 250 points and terminated the task. The odds of losing, indicated by number of bomb cards (ranging from 1 to 5), varied across trials. In this task, the number of cards chosen served as an index of risk-taking. Information about the number of gain and bomb cards, as well as the points associated with gains and losses, was displayed at the top of the screen for each round. As expected, participants paid attention to these features as they influenced their solo choices: the number of selected cards decreased as a function of the number of loss cards and the total number of cards in the trial (see Supplemental Materials, Fig. S1). These different features were factorially crossed to create two unique but comparable sets of 10 trials, a necessity for the two source conditions within the social version of the task. This set of 20 unique trials resulted from a pilot study (*N* = 73, aged 16 to 18 years), showing enough choice variability without ceiling and floor effects in participants’ choice data on these trials. This was a prerequisite for designing the social version of the task that relied on presenting real peer choices that were collected in session 1 from the participant’s classmates. In the CCT, participants selected cards by clicking on them, which were then marked grey (see Fig. 2). In this game, cards were not immediately revealed, so participants did not know whether they encountered a gain or loss card (a design known as the ‘cold’ CCT; Figner et al. 2009). This design choice eliminated experiencing the outcome of one’s choice, to rule out learning effects that might lead to a change in risk attitude in a follow-up session of the task.

Before starting the task, participants completed two practice rounds. During these rounds, cards were revealed one by one, and programmed as such that participants encountered a bomb card once quickly and once after several gain cards. Participants were instructed to maximize their points and could only proceed after correctly answering test questions about the task. Three randomly selected trials were played out at the end of the game. Thus, participants only received feedback about the number of points earned based on 3 random trials. Points collected in these trials were converted into lottery tickets for a classroom raffle that could result in winning a gift voucher of 40 euros. The game was programmed and administered using NeuroTask (NeuroTask Scripting Beta, https://scripting.neurotask.com/).

#### Identifying Friends and Popular Peers in Class

**Fig. 3:**
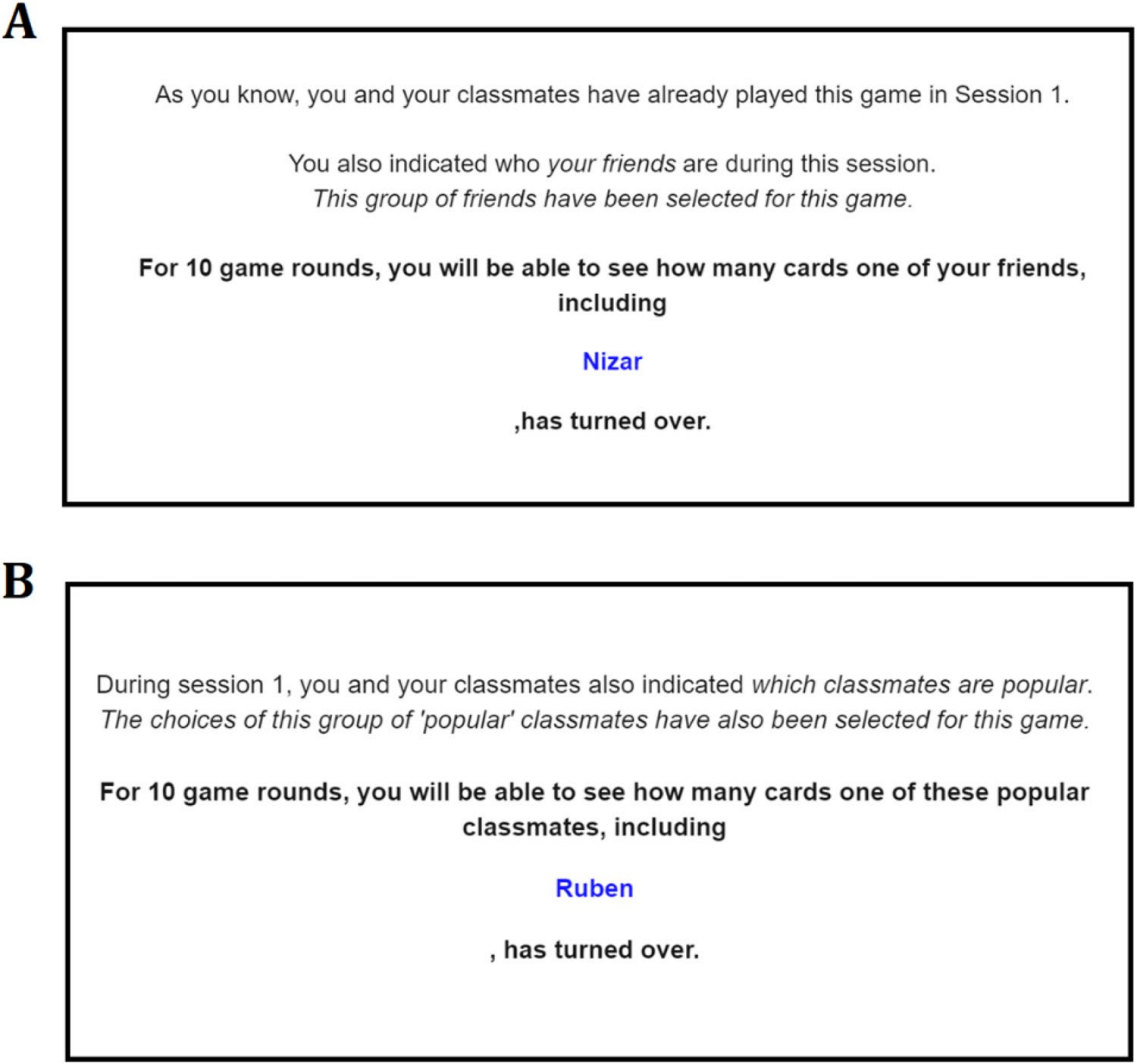
Introduction screen for the social source ‘friends’ (A) and ‘popular’ classmates (B). The two social source conditions were introduced as groups without displaying individual names. To enhance the credibility of the manipulation and guide adolescents in imagining the sources, an example name was provided.

Participants completed a peer nomination questionnaire to determine which classmates were their friends (with the items *‘who is your friend’* and *‘who is your best friend’*) and which peers were perceived as most popular by the participant (with the item *‘who is most popular?’)*. The choices of these peers were presented to the participants in the social version of the CCT. These peers were not introduced by their real names but were instead identified by the group to which they belonged *(“your group of friends*” and *“the group of popular classmates”*). To enhance the credibility of manipulation, an example name was provided in the instructions (see Fig. 3). For each nomination item, participants could select as many names as they wanted or the option ‘nobody’. This resulted in binary coding (1 = yes, 0 = no) for each classmate dyad (participant - classmate X), indicating whether the classmate was nominated for a certain characteristic by the participant.

Participant’s individual popularity nominations and the class-level nominations were both used to select the real-made choices of peers that could be presented in the ‘popular peer’ condition of the social CCT. Peers were considered popular at the class level if they received the 5 highest number of group nominations for the *‘who is most popular?’* item. This was based on all the nominations provided by the participating pupils in class at session 1.

In addition to the abovementioned items, participants nominated their classmates for several other items. Previous literature suggests that the (behavioral) profile of popular adolescents is quite heterogeneous and partially dependent on peer culture (Cillessen & Marks, 2011). Therefore, these nomination items were used to provide additional background information on other characteristics of this group within the current context. See Table S1 in the Supplemental Materials for the full list of nomination items that were presented to the participants.

#### Social Version of the Columbia Card Task: Measuring Choice Adjustments

**Fig. 4:**
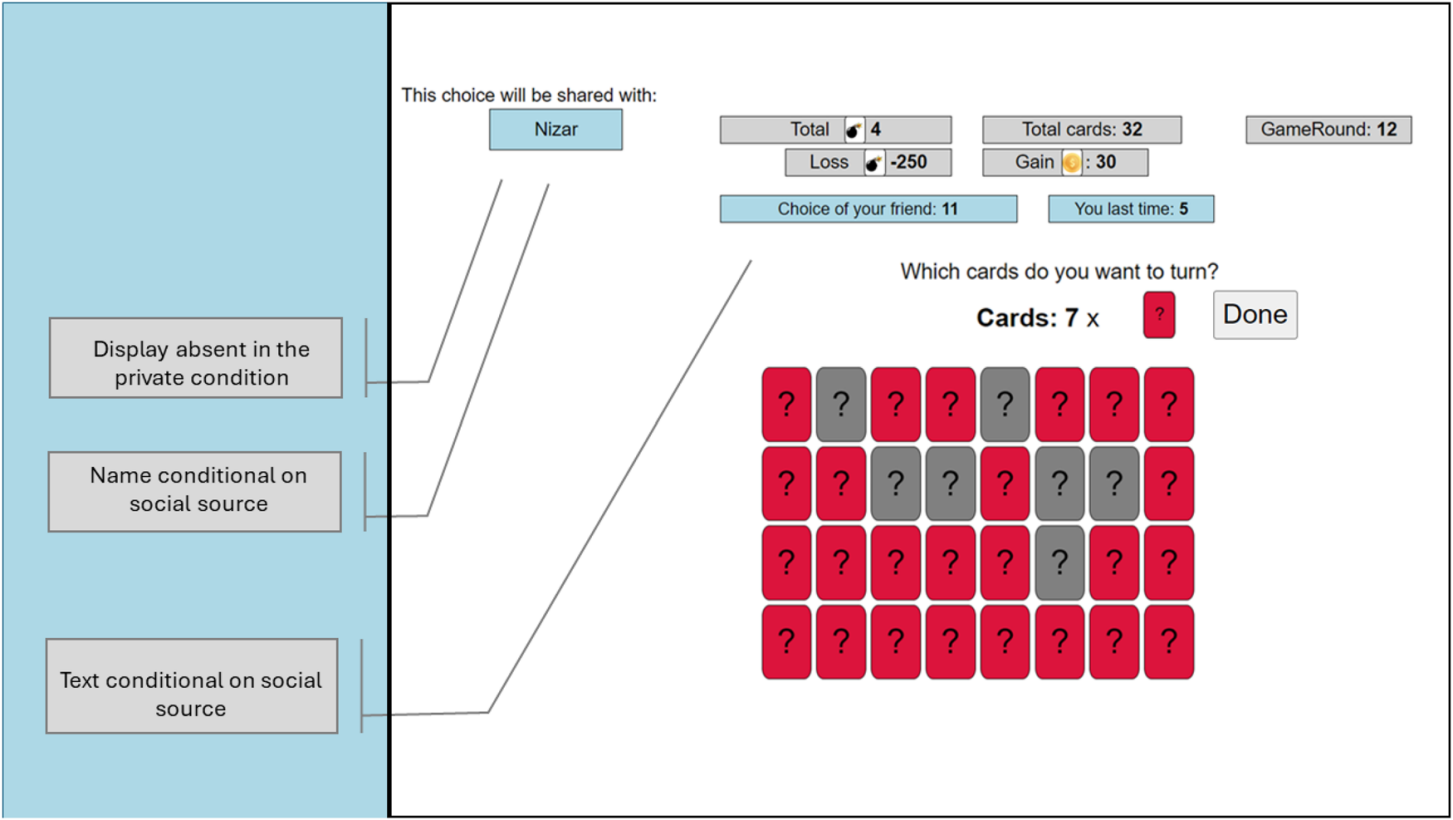
Decision screen from the Social version of the Columbia Card Task. In session 2, participants played a new version of the CCT, where they were informed about the choice made by a popular peer or friend from session 1. They were also reminded of their own choice for that specific trial. Participants in the observation condition were informed about who would be observing their choice, as indicated in the left corner of the screen in each trial. The observer was either a peer nominated as a friend or a peer nominated as popular, depending on the social source condition. This left corner display was absent in the private condition.

In the second session, participants played a social version of the CCT to examine how observing the choices of popular peers and friends affects adolescents’ decision-making. This version mirrored the solo version but included the addition of showing the number of cards selected by either a friend or a popular peer for each round (See Fig. 4). The screen also displayed the number of cards the participant had selected in the previous solo version for that specific trial, to remind them on their previous choice, and thus making possible adjustments more explicit for the participant (see Fig. 4). Participants were informed though a pop-up about which social source would provide information for the subsequent 10 trials (see Supplemental Materials, Fig. S2, screen 3). During each trial, participants were presented with a choice from a single friend or popular peer (see Fig. 4).

#### Introducing the Social Sources to the Participants

During the instructions, participants were informed that they would view choices from two groups: *‘a group of friends’* and *‘a group of popular classmates’* (see Fig. 3). These sources were introduced as groups to provide more flexibility in selecting which specific real-made choices participants would observe from each source (see Supplemental Materials, ‘Preparation of the Social Information’ for details). To enhance the credibility of the manipulation, an example name was provided for each source (i.e., friend name and a popular peer name, see Fig. 3). Example names for the friend group were chosen based on the criteria of being identified as a best friend and, if possible, being gender-matched (Gaughan, 2006). In cases where data of a best friend was unavailable, a gender-matched friend was selected instead. For the popular peer group, a name was presented from a peer that was nominated by the participant as being popular, and/or that belonged to the top 5 popular peers in class, based on the total popularity nominations received from the class. For selecting the source, a preference was given to popular peers that matched gender with the participant and was perceived as being most popular by the participant. 87% of instances, successfully presented participants with an example peer name that matched to their gender.

#### Features of the Private and Observation Condition

Participants assigned to the observation condition were informed during the online instructions that their final choices would be shared with two classmates. Names of these students were displayed, which presented a peer nominated as friend and a peer nominated as popular (see Fig. S2-Screen 4 C). In the observation condition, the example names of the peers introduced earlier (see Fig. 4) were also selected as the observing peers. Per trial, a display showed which of those two peers would observe their choices (see Fig. 4); In the friend condition, a friend’s name was shown, whereas in the popular condition, a popular peer’s name was displayed. These names were kept stable across the friend- and popular peer-trials.

#### Disclosing a Profile Screen in the Private and Observation Condition

Participants were led to believe that, at the end of the game, a profile screen would be shared with these observing peers, indicating the number of cards both the participant and peer dared to turn over in each round, along with a bar visualizing how much their behavior overlapped (see Supplemental Materials, Fig. S2- screen 4). At the start, participants were also informed about whose choices they would view at the end of the game (see Supplemental Materials, Fig. S2, screen 5) to reinforce their belief that their own choices would be disclosed. In reality, participants’ choices were shared with two randomly selected classmates, and they were debriefed about this matter at the end of the experiment. In the private condition, participants’ final choices remained confidential. To match the observation condition, participants were informed that they would see a profile screen as well, but this screen only displayed the participant’s average number of selected cards (see Supplemental Materials, Fig. S2-screen 6).

#### Preselected Peer Choices in all CCT Conditions

All displayed choices were preselected based on the distance between the participant’s initial choice and the peer’s choice, with differences ranging from 3 to 20 cards (see Fig. S7 for a distribution of these distances). Thus, participants were able to respond on peer information that deviated from their initial response. For more details on the selection procedure, see section ‘Preparation of the Social Information’ of the Supplemental Materials.

### Statistical Analysis

#### Social Adjustment (s) in Response to peers’ Choices

The primary dependent variable was social influence on the number of cards selected during each CCT trial. Social influence was measured by calculating the relative adjustment of participants’ initial choice (i.e., selected cards) in the direction of social information (SI; Molleman et al., 2019). This adjustment score for each CCT trial was calculated using the following formula:

$$\:s=\:\frac{{C}_{2}-{C}_{1}}{SI-{C}_{1}}$$

Here, the difference between participants’ second choice (C1) and first choice (C2) was computed and divided by the difference between the provided social information (SI) and the participant’s first choice (C1). It was expected that most participants would either ignore, copy or move toward social information, resulting in values between 0 < = s < 1. Moving away from the SI (anti-conformity; s < 0) or changing the initial choice beyond the SI (overshooting; s > 1) were also seen as plausible responses. Therefore, as documented in our preregistration (https://osf.io/5ma8x/?view_only=4f27c0d802354de1a7477deec2a21937), all possible values for s were included in the analysis.

#### Assessing the Dependency of Social Adjustment(s) on Social Source and Social Condition

A linear mixed-effects model was performed to predict social adjustment (s), including a random intercept at the participant level to account for individual differences. The model included an interaction effect between social source and decision context to examine whether the influence of popular peers was greater in the observation condition and whether friends had more impact than popular peers in the private condition.

As second step, the direction of social information (i.e., whether it indicated turning over more or fewer cards) was included as a covariate to account for its potential impact on choice adjustment (Braams et al., 2019; Chung et al., 2015; Reiter et al., 2019). Although our selection procedure aimed to minimize differences in the type of social information presented between the two source conditions, some minor variations were inevitable due to the use of real social information. Nevertheless, an inspection of the distribution of social information direction received by participants within each source condition and across the two contexts revealed no specific bias (see Supplemental Materials; Fig. S5 and S6).

#### Exploratory Age Analysis

As the literature provides no clear understanding of the expected age dependency for the impact of both social sources on choice adjustment, a linear and curvilinear age term was included in interaction with the source type (Cohen & Prinstein, 2006; Field et al., 2023, but also see: Pinho et al., 2021; Slagter et al., 2023b). Age was standardized to improve interpretability of the interaction coefficient and to reduce multicollinearity between the linear and nonlinear age terms.

#### Correcting for Overlap Between Peers Perceived as Friend and Popular

As in practice, the popular group and friend group of adolescents cannot always be seen as complete pure, isolated groups. As the experimental paradigm examined the influence of existing sources, it was expected that the two information sources were not always completely pure. Therefore, a contamination score was calculated for each participant that would reflect the extent to which a participant might think of the other source category as well, when facing the choice of a friend or a popular peer. While choices were explicitly communicated as being the choice of a friend or a popular peer, participants might still partially think of popular peers or friends, respectively, if in practice they viewed (some) friends as popular or vice versa.

For each participant and source condition a contamination score was calculated by the following formulas:

$$\begin{array}{lll}Overlap\:f\to\:p=\displaystyle\frac{Number\:of\:friends\:nominated\:as\:popular\:}{\begin{array}{l}Number\:of\:friend\:nominations\\\qquad \left(friend\:group\right)\end{array}}\\\\\qquad if\:source\:condition=friend\end{array}$$

$$\begin{array}{lll}Overlap\:p\to\:\:f=\displaystyle\frac{Number\:of\:popular\:peers\:nominated\:as\:friend\:}{\begin{array}{l}Number\:of\:popular\:peers\:nominations\\\qquad\left(popular\:peer\:group\right)\end{array}}\\\\\qquad if\:source\:condition=popular\end{array}$$

$$\begin{array}{lll}Contamination\:score\:\left\{\begin{array}{lll}Overlap\:f\to\:\:p\:,\:if\:source\:condition\:=\:friend,\\ Overlap\:p\:\to\:f,\:if\:\:source\:condition\:=\:popular\end{array}\right.\end{array}$$

This score has a value between 0 and 1 and reflects the fraction of the individuals shown in the assigned condition who also belonged to the other category. This can be seen as an indicator of how “pure” the assigned source was, with a value of zero indicating zero contamination. For example, a value of 0.25 for the friend condition would indicate that the 25% of the friend group were also seen as popular by that specific participant. In other words, one could argue that the source condition is contaminated with the other category for 25%. Indeed, classmates were sometimes seen as friends and popular as well. The level of contamination differed between participants and the two sources. See Fig. S8 for the distribution of contamination scores, which range from 0 to 1, with a median of 0. This figure illustrates that the popular group had more often higher contamination scores (M = 0.27, SD = 0.37) than the friend group (M = 0.16, SD = 0.24), but for most participants both source conditions were constructed without any contamination.

These individual contamination scores for each source condition were added as a covariate in all regression models to correct the comparison between source conditions for possible contamination that might have influenced our primarily detected source effect. Additionally, a sensitivity analysis was performed by excluding all participants who had a contamination score above 0.4 for one of the two source conditions (*N* = 150).

## Results

### Additional Information on How Popular Peers and Friends were Perceived

In this sample, popularity nominations were most strongly associated with perceptions of classmates as ‘influential’ and ‘leaders’. Conversely, friendship nominations were most strongly associated with perceptions of peers as ‘trustworthy,’ ‘cool’, and ‘liked’, which showed a weaker association with popularity nominations (see Fig. S3). Only a small group of popular peers were perceived as mean or unkind (Fig. S4), indicating that only a small proportion of popular peers had a negative valence.

### Adolescents Adjust Their Choices Slightly More in Response to Friends Than to Popular Peers

**Fig. 5:**
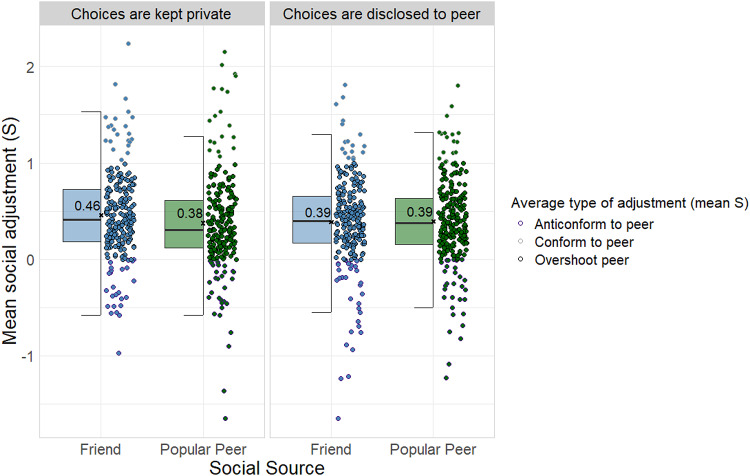
Social adjustment across the different decision contexts and social sources. This figure presents the mean social adjustment value per participant for each social source and context (private choices versus disclosed choices to the peer). Dots, representing the mean social adjustment (s) of each individual, are color-coded to reflect the individuals’ average type of adjustment. Group-level mean values are represented by labeled black crosses.

On average, adolescents adjusted their choices (M = 0.40, SD = 1.05, range= [-7.5 -7.5]) in 88% of the cases in response to their peers’ choices. There was a significant main effect of social source, with adolescents adjusting slightly more to friends’ choices than to popular peers’ choices (β=−0.08, 95% CI [− 0.14, − 0.01], *p* = 0.018, see Table 2, model 1). The main effect of the observation condition was not significant (β=−0.06, 95% CI [− 0.15,0.02], *p* = 0.151), suggesting that the decision context did not have a strong influence on the dependent variable. The interaction between social source and observation condition approached but did not reach statistical significance (β = 0.08, 95% CI [− 0.01,0.17], *p* = 0.074), suggesting that the effect of social source on social adjustment may vary by decision context, but was too small to detect in this sample size. Figure 5 illustrates the participants’ mean adjustment value (S) across social sources and decision context.

### Adolescents Adjust Their Choices More When Facing Riskier Choices Than When Facing Safer Choices

**Fig. 6:**
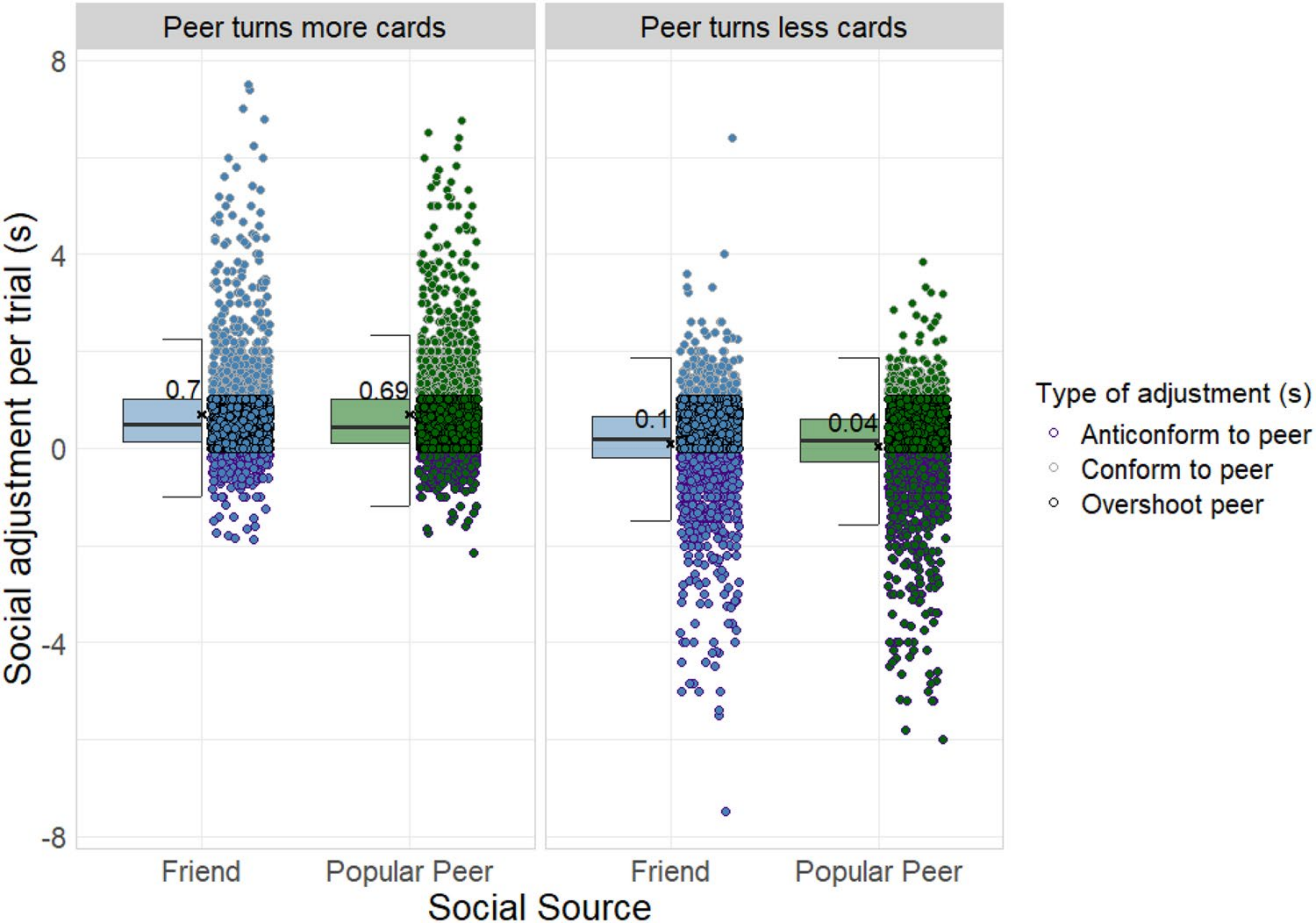
Social adjustment depends on the direction of peer choice. The figure illustrates social adjustment values per decision trial, categorized by the direction of peer choice received (more or fewer cards). Each dot is color-coded to indicate the type of adjustment shown in each decision trial. Group-level mean values are denoted by labeled black crosses.

As a follow-up, it was examined whether participants’ adjustment tendencies were influenced by the direction of the observed peer’s choice (i.e., more cards or fewer cards were selected by the peer). It was anticipated that the direction of social information would be an important covariate for the model, as adolescents have been shown to respond differently to safe and risky peer choices (Braams et al., 2019; Reiter et al., 2019). Indeed, adjustment values depended on the direction of the peer’s choice (see Fig. 6). Participants adjusted their choices more when observing risky peer choices than when observing safer peer choices (B= -0.65, 95% CI [-0.70 – -0.60], *p* < 0.001; see Table 2, Model 2). More specifically, there was a greater shift in the number of cards (M = 4.84, SD = 6.46) when participants observed more risky choices compared to safe choices (M= -0.87, SD = 7.12; see Supplemental Materials Fig. S9). 

### Social Adjustment is Similar Across Ages, Irrespective of the Source

The confirmatory models were followed up with an exploratory model including linear and quadratic age effects to investigate whether the impact of the source was moderated by age. The results showed that the difference in impact between sources did not change with age (see Table 2, Model 3), and no main effect of age was observed. After accounting for age differences within our sample, the effect of social source was found to be nonsignificant. In addition, as preregistered, analyses controlled for the nonmatch in gender between the presented social source and the participant (this was only the case for 13% of the participants). This did not affect the results (see Supplemental Materials, Table S3).

### Results Hold When Correcting for Individual Contamination of the Social Source Condition

A covariate was added to correct for cases where the social source conditions could not be considered completely distinct. Impurity was calculated for the friend and popular peer condition, based on how many individuals within these peer groups were also seen as the other category (i.e. friend or popular). These impurities, referred to as contamination score (see Fig. S8 for a distribution), were added as a covariate to the model. Correcting for this contamination did not change the results of any of the primarily models (see Supplemental Materials, Table S4). In addition, a sensitivity analysis was performed by removing 150 participants for which one of the source conditions had a contamination score above 0.4, meaning that 40% of the popular peers were also seen as friends by the participant. The estimated effects of the model remained robust (see Table S5).

**Table 2:** Predictors of participants’ social adjustments (S) within the Columbia Card Task

Predictors	Model 1	Model 2		Model 3	
	β (95% CI) *p*-value		β (95% CI) *p*- value		β (95% CI) *p*- value
Intercept	0.45 (0.39–0.51) *p* < 0.001		0.74 (0.68–0.80) *p* < 0.001		0.75 (0.67–0.82) *p* < 0.001
Social source [popular]	-0.08 (-0.14 – -0.01) *p* = 0.018		-0.07 (-0.13– -0.01) *p* = 0.026		-0.06 (-0.13–0.02) *p* = 0.151
Observation condition [yes]	-0.06 (-0.15–0.02) *p* = 0.151		-0.03 (-0.11–0.05) *p* = 0.465		-0.03 (-0.11–0.05) *p* = 0.436
Social source [popular] × Observation condition [yes]	0.08 (-0.01–0.17) *p* = 0.074		0.07 (-0.01–0.16) *p* = 0.101		0.08 (-0.01–0.16) *p* = 0.086
Direction [fewer cards]			-0.65 (-0.70 – -0.60) *p* < 0.001		-0.65 (-0.70– -0.60) *p* < 0.001
Age scaled (continuous)					-0.01 (-0.05–0.03) *p* = 0.672
Age scaled (Quadratic)					-0.01 (-0.05–0.04) *p* = 0.768
Age scaled (continuous) x Social source [popular]					0.02 (-0.03–0.06) *p* = 0.479
Age scaled (Quadratic) x Social source [popular]					-0.02 (-0.03–0.06) *p* = 0.512
Number of participants (N classes)	457 (35)		457 (35)		457 (35)
Number of observations	7562		7562		7562

Note. Linear mixed-effects models were fitted to choice adjustments, using ‘participant’ as a random intercept. Model 1 tested whether participants’ choice adjustments were influenced by the social source (popular peer versus friend) and whether this effect was dependent on the social condition (observation versus private). Model 2 included the direction of the peer choice (fewer cards versus more cards) as a covariate to test whether participants’ choice adjustments were influenced by direction. Exploratory age effects were tested in Model 3. The estimated effects are presented with 95% confidence intervals (CIs) in parentheses and P values below the CIs

### The Response Strategy Depends on the Direction of The Peer’s Choice

**Fig. 7:**
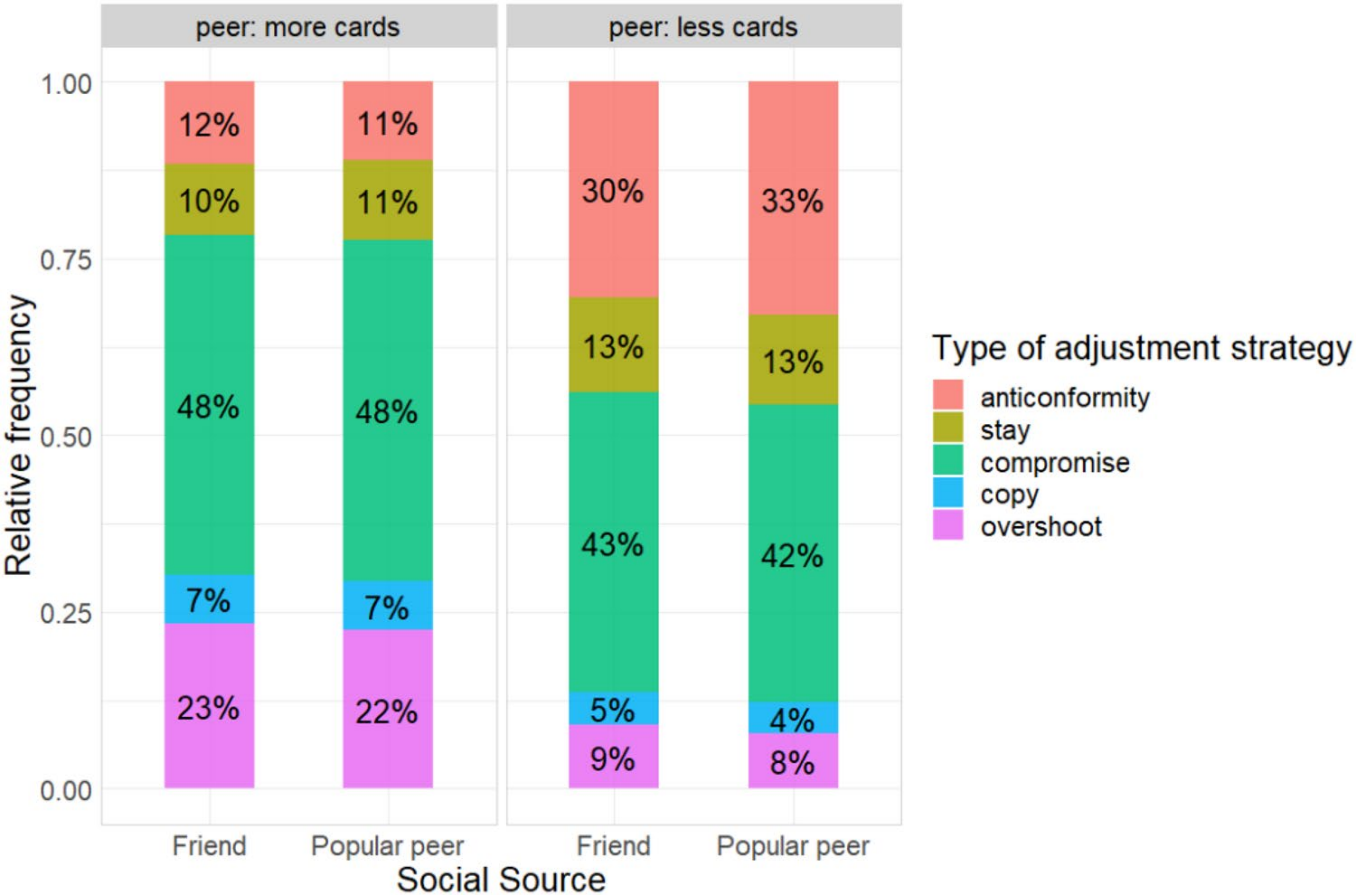
Adolescents’ choice adjustment strategies in response to observing their peers’ choices. The relative frequencies of strategies applied by adolescents in response to the peer’s choice are displayed. These frequencies are plotted for both social sources (friends or popular classmates) and separated based on the direction (riskier or more cautious choice) of social information. The frequency of anti-conformity and overshooting is highly dependent on the direction of the social information, irrespective of the source. Visual inspection suggested no clear differences between the relative frequency of strategies employed by adolescents across social sources.

To gain a deeper understanding of adolescents’ responses to social information, it was examined which strategies they used to incorporate this information into their initial choices through descriptive statistics. Figure 7 illustrates these response strategies, comparing adjustments in response to the choices of a friend versus a popular classmate, and examining how these responses vary based on the direction of the social information.

In about half of the cases, adolescents chose to compromise, where their final choice fell between their initial choice and the peer’s choice. Approximately 10% of the time, participants maintained their initial choice, a pattern consistent for both friends and popular classmates. The rate of copying another’s answer was also similar for both sources.

Strategy differences did emerge when considering the direction of the information. When adolescents observed a peer selecting more cards, they were more likely to copy this choice (7% compared to 4%) or to exceed the peer’s choice (22% compared to 8%) than when observing more cautious behaviour (fewer cards). In situations where peers took less risk by turning over fewer cards, adolescents displayed anti-conformity in 33% of the cases, by turning over even more cards. This anti-conformity was much less frequent in response to riskier choices (11% compared to 33%).

## Discussion

To understand how adolescents’ risk behavior is influenced by their real social network, it is essential to identify and understand which peers serve as a source of influence. This study investigated the influence of friends and popular classmates on adolescents’ risky decision making. Adolescents’ tendency to adjust their choices was measured in a risk-taking game after they observed the choices made by these two types of sources. As different motives to adjust one’s behaviour are at play in a private and public context, this study investigated the relative influence of these sources in both settings. The results showed that adolescents were influenced by both sources, with friends being slightly more influential. The degree of influence from the two sources was the same in both social contexts, regardless of whether the choices were shared or not shared with their friends or popular classmates. Exploratory, this study found that risky choices appear more appealing than safe ones; adolescents were willing to surpass their peers’ risky decisions while ignoring safe peer choices more frequently.

Within the private context, a small difference effect on social adjustment was found between friends and popular peers, in favor of friends. These results provide a better understanding of the magnitude of influence of popular peers that were previously mainly compared to nonpopular (unknown) peers (e.g., Cohen & Prinstein, 2006; Teunissen et al., 2012). These findings, found in the private context, suggest that adolescents adjusted their choices to maintain a sense of belonging (Baumeister & Leary, 1995; Laursen & Veenstra, 2021) and/or experienced preference uncertainty (Moutoussis et al., 2016). If so, these motives drove adolescents to be slightly more influenced by their friends compared to popular peers, which might relate to a greater feeling of social identification (Analytis et al., 2018; Laursen, 2017; Trivers, 1971). However, popular peers showed to be almost as influential as friends, supporting the notion that they are also a significant source of influence when adolescents are confronted with their behaviour. While this study suggests that friends will likely exert the greatest direct effect on risk behavior at the individual level, this may affect the group norm to a lesser extent than popular peers who show to have influence beyond their friendship connections. These results suggest that targeting popular peers for classroom interventions aimed at behavioral change or improving class cohesion might be even more effective and low-cost than targeting individual friends (Paluck et al., 2016).

The absence of an effect of observation for both peer sources might seem to contradict the findings of numerous studies that showed behavioral changes as the result of peer observation (e.g., Chein et al., 2011; Somerville et al., 2019; Defoe et al., 2020; Gardner & Steinberg, 2005). However, these studies compared adolescents’ choices made alone, to their choices made while being monitored by another peer, without providing explicit information about the choice preference of the peer that was monitoring. In our study, observing the choices of familiar peers, which had a significant impact in a private context, might have trumped the smaller additional effect of peer presence. In line with this, studies with a more similar setup (Haddad et al., 2014; Harakeh & de Boer, 2019) showed that explicit advice or seeing the choices of other peers have a greater influence on social adjustment compared to mere peer presence. In addition, a recent meta-analysis showed that the effect of peer presence is rather small and depends strongly on whether the participant knows whether the observing peer is risk neutral, pro-risk or anti-risk (Powers et al., 2022). Future studies could compare decision contexts where adolescents receive peer advice on screen to those where they are also directly monitored by the peer providing advice. When peer information is framed as advice and peers can directly monitor its’ adherence, this might induce a greater fear of peer rejection and consequently a stronger effect of peer observation.

It was found that adolescents’ tendency to adjust their choices was greater when they observed peer choices that were riskier than their initial choice. Exploring the adopted adjustment strategies revealed that this effect was mainly driven by adolescents’ tendency to overshoot the number of cards turned over by the other peer, while showing more frequently anti-conformity when observing a safer choice. Thus, adolescents are more likely to reject cautious behavior and are more inclined to play even riskier compared to their peers. This pattern is in line with, but also extent, studies that showed that adolescents conform more toward risky choices or perceptions than toward safe choices or perceptions (Reiter et al., 2019; Knoll et al., 2017) and those showing resistance to safe peer advice (Haddad et al., 2014; but see: Braams et al., 2019). Adolescents’ greater sensitivity to risk information might be linked to their belief that such behaviour signals competence and strength (Ellis et al., 2012; Salas-Rodríguez et al., 2023). Confronting adolescents with their peers’ choices might lead to social comparison (Festinger, 1954), as people naturally compare themselves to others to identify their strengths and evaluate their performance. Thus, adolescents might have increased their risk-taking to feel braver or tougher than their peers, contributing to a positive self-concept and self-image. Supporting this idea, by qualitative studies found that being “cool and tough” is one of the mentioned motives for adolescents to engage in risk-taking (Defoe et al., 2022; Littlecott et al., 2023). Overall, our results show that adolescents considered the direction of social information, indicating that the observed adjustment was not an unconscious automatic imitation process (Chartrand & Bargh, 1999). In addition, these results highlight the importance of studying overshooting and anti-conformity tendencies. In real-life situations, when multiple interactions occur between dyads of peers, overshooting could lead to escalation of risky behaviors. Risk taking might increase incrementally at the group level as adolescents push limits in response to others (e.g., skipping class more frequently, greater damage of property such as a bus shelter), and subsequently, this might shape new perceptions about descriptive norms (frequency of certain behaviors) and injunctive norms (appropriateness of a certain behavior) in favor of (new) risk behavior. Together, the results suggest that when adolescents are exposed to the behavior of friends and popular peers, they may adjust their actions based on internal motivations such as doubt and the desire to make optimal decisions, the need to feel more connected with peers they like, or the urge to outperform others. This may not only apply to risky behaviors, as observed in this study, but also to other behaviors such as prosocial actions and health-related activities (like exercising and dietary habits).

### The Strengths and Specific Context of the Findings

This study quantified and compared adolescents’ behavioral adjustment in response to the choices of friends and popular peers. The experimental paradigm, using real information from familiar peers, enabled the investigation of the influence of these two types of peer sources within adolescents’ own ecological context. Compared to non-experimental work, this study was able to isolate and measure the direct influence of peer exposure on behavior, leading to less noisy data and limiting undetected confounding effects, while focusing on existing sources. Such a highly controlled yet naturalistic social setting contributes to reliable estimates of peer effects. However, the findings should be interpreted in a specific context. Below is a specification of how the results can or cannot be generalised to different contexts. First, our results pertaining to the influence of popular peers might be specific to the demographics of our sample, which consists of Dutch high school students (aged 11–18 years) who follow pre-university and preparatory education. Previous literature shows that popular peers are associated with a variety of profiles that are peer culture dependent, affecting how the concept of popularity itself is perceived (Li et al., 2012; Closson, 2009; Peeters et al., 2021; Postigo-Zegarra et al., 2021; Zhang et al., 2018). Therefore, the influence of popular peers might differ when assessed in a sample with a different peer culture and hierarchy. This study measured the influence of perceived popular peers without distinguishing between subtypes of popular peers, such as prosocial and antisocial individuals, who differ in their level of prestige and dominance (Cheng et al. 2013; De Bruyn and Cillessen 2006a; Bruyn and Cillessen 2006b; van den Berg et al. 2015). In this study’s sample, it turned out that more than half of the popular peers were also liked, and only a small group of popular peers were perceived as mean or disliked. Therefore, the influence of popular peers should not be generalised to other peer cultures without having clear understanding of whether these popular peers are rather liked or disliked. Lastly, our results should not be confused with or generalised to other forms of social status, like sociometric popularity (Cillessen & Marks, 2011).

Secondly, this study used an abstract risky decision-making task, to be practically and ethically able to show real choices made by friends and popular peers (i.e. instead of looking at smoking offers and smoking tendencies). This new abstract task also reduces confounding variables such as prior beliefs, experience and knowledge that can affect susceptibility to social influence. However, researchers must exercise caution when directly translating these results to more complex risk behaviours that already carry a strong social norm or for which adolescents may have a strong preference due to prior experience and knowledge (Moutoussis et al., 2016; Reiter et al., 2021). These additional factors may influence whether an individual is more or less likely to conform to the choices of others. Additionally, the stakes in this game are not as meaningful as real-life risks (e.g., financial ruin or physical harm), which may lead to greater tendencies to surpass other’s risky behaviour than for more harmful risky behaviours. While there is uncertainty about how this would extrapolate to other risky behaviour, the discussed factors are believed to mainly affect one’s overall willingness to adopt or surpass peer choices in general but would not affect the small difference in effect found for popular peers and friends when making predictions for other risk behaviours. At very least, it can be stated that these findings can be applied to a context where behaviours involve limited harmful risks, and where adolescents do not yet have a strong preference regarding how they want to act.

### Future Directions Addressing Limitations

These findings raise new questions that future studies could address in order to better understand the results and the broader context to which they can be applied. First, in real-life situations, adolescents receive information about peer behavior from different sources simultaneously and multiples times a day. Our paradigm can be extended by presenting peer information from multiple sources simultaneously (see also Kwon et al., 2021). Such a design could be used to study what happens when popular peers need to compete with conflicting information expressed by friends. This would indicate whether the influence of popular peers persists when other sources (offering opposing advice) are available. Another way to extend the paradigm is to add multiple observations over time, which would allow us to study the cumulative effect of multiple observations, and investigate the potential escalation of risk behavior due to adolescent’s tendency to surpass others. Second, our data reveals individual differences in the main response strategy that individuals apply. Adolescents did not only show compromise and copy tendencies, but also tendencies to overshoot risk and anti-conformation toward safe choices. This suggests that multiple motives for adjustment are plausible and may vary across individuals, leading to the use of different social adjustment strategies. Within the private context, copying and compromising is likely driven by (induced) uncertainty or the motive to maintain once’s sense of belonging. However, showing off more risky behavior rather links to boosting self-concept through social comparison. To further disentangle this dual motivation of standing out and fitting in (Gray, 2017), and the role of uncertainty, future studies should also include additional measures to capture adolescents feelings and experiences before and after exposure to peer behavior. These measures should focus on perceived toughness, sense of belonging and choice uncertainty. This could be combined with qualitative data on adolescents’ own self-generated motives for adjustment (Defoe et al., 2022) to confirm our suggested motives. Thirdly, future studies should investigate whether our results extend to other subtypes of popular peers, such as prosocial and antisocial individuals, within various peer cultures (Cheng et al. 2013; Closson 2009; De Bruyn and Cillessen 2006a; Bruyn and Cillessen 2006b; Li et al. 2012; Peeters et al. 2021; Postigo-Zegarra et al. 2021; van den Berg et al. 2015; Zhang et al. 2018). The influence of antisocial or disliked popular peers may be minimal in private decisions but might be comparable to or even greater than that of friends in public contexts if these types of intimidating popular peers are present. Finally, an important next step is to investigate the long-term effects of friends and popular peers. On the long-term, differences in impact will increase if peer behavior from both sources is not equally internalized. By investigating whether information from popular peers and friends influences adolescents’ risk attitudes, one can better predict whether these peers have long-term effects, even if they are no longer exposed to their behaviour in the future (cf. Cohen & Prinstein, 2006).

## Conclusion

To understand how adolescents’ risk behavior is influenced by their real social network, it is essential to identify and understand which peers serve as a source of influence. Previous work identified the role of friends and popular peers, but little is known about their relative influence in a public and private context. This study compared the influence of friends and popular classmates on adolescents’ risky decision making, with and without disclosure of their choices to these peers. In conclusion, this study shows that adolescents are sensitive to the choices of friends and peers perceived as popular and kind, regardless of whether these choices are disclosed to these peers. The high tendency to adjust choices without disclosure, for both sources, suggests that uncertainty in choice preference or the need for a sense of belonging are key motives for adolescents to alter their risk choices when observing the behaviour of these familiar peers. Friends had only slightly more influence than popular peers in both conditions. Further research should determine whether this pattern holds only when the popular adolescent is also perceived as likable, as was predominantly the case for the popular peers in this study. Lastly, this study illustrates that adolescents respond to peer behaviour through various strategies: copying, compromising, anti-confirming, ignoring or overshooting. The strategy depended on the type of behaviour displayed by their peer: adolescents were more willing to go along with risky behavior than safe behavior and even felt compelled to surpass others’ risky actions. This implies that risk behavior is also evoked by adolescents’ need to feel braver and tougher than their familiar peers. This study advances experimental research on peer influence by demonstrating that allowing for and capturing different adjustment strategies is crucial. These nuances provide a better understanding of adolescents’ sensitivity to various social influences and the motivations behind it, which is crucial for developing effective health promotion interventions.

## Supplementary Information

Below is the link to the electronic supplementary material.

Supplementary Material 1

## Data Availability

All data and code supporting the findings of this study are made available on the first author’s OSF page: https://osf.io/6a5dp/?view_only=8fbb5b734d5d430590498564e924134b.
