# Supplementary material for: An Experimental Study on the Influence of Friends and Popular Peers on Adolescents’ Risky Decisions in a Private and Public Context
Source: J Youth Adolesc. 2026 Apr 15;55(8):1964–80. doi: 10.1007/s10964-026-02349-2 (PMC13442566; doi:10.1007/s10964-026-02349-2)
Supplement: Supplementary file 1 [file 10964_2026_2349_MOESM1_ESM.docx]

# **Supplementary Materials:**

**An Experimental Study on the Influence of Friends and Popular Peers on Adolescents' Risky Decisions in a Private and Public Context**

# **Contents**

1. Supplementary Figures

2. Supplementary Tables

3. Supplementary Methods

**Supplementary Figures**

**Figure S1**

*Group-level sensitivity for the variability in CCT characteristics*

*Note.* As expected, the features of each play round influenced participants’ trial-by-trial decisions in the solo version of the task, which suggests that participants paid attention to the specific features of each play round. The number of cards selected for turning decreased as a function of the number of loss cards. Moreover, an overall increase in the number of cards selected for the 40-card trials compared to the 32-card trials was observed. Participants did not seem to be sensitive to the small difference in gain amount.

**Figure S2**

*Screen 1- Introduction social source ‘friends’*

*Screen 2- Introduction social source ‘popular peers’*

*Screen 3 – Introducing the social source for the first and/or second set of 10 rounds.*

*Screen 4A – Introduction profile screen of observation condition, instruction page 1*

*Screen 4B – Introduction profile screen of observation condition, instruction page 2*

*Screen 4C – Introduction profile screen of observation condition, instruction page 3*

*Screen 5 – Introducing the peer choices that the participant will observe*

*Screen 6A – Introduction profile screen in the private condition*

*Screen 6B – Introduction profile screen in the private condition*

*Note*. This figure presents a subset of the instruction screens from the social version of the task, demonstrating how the social source condition and decision context (private versus public) were introduced to the participant. The task was administered in Dutch, and all screens have been translated into English for illustration purposes.

**Figure S3.**
*Strength of association between peer nominations given by the participant to their classmates*

*Note.* The figure illustrates the overall strength of association (Cramér's V) between the peer characteristic variables across the entire dataset. Thus, it represents global associations scores, summarizing the strength of the relationship across all categories in a contingency table, instead of presenting conditional correlations or probabilities (see Fig. S4). Strength of association is based on the data (N observations =11964) of the participants who participated in session 2 and were included in the main data-analysis (N = 457). The heatmap codes the Chi-square P-value.

**Figure S4**

*Note.* A) Categorizes all the ‘most popular peer’ nominations given by the participants (N _nominations_ =1499) based on other nomination (i.e. characteristics) that were given to these popular peers. Most peers nominated as "most popular" were also nominated as someone they liked (45.6%) or were not explicitly mentioned as being liked or disliked (30.5%). Only 15.9% of the popular peers were seen as unkind and/or mean, also a small group of the popular peers was admired (7.5%). B) Categorizes all the ‘friend’ nominations given by the participants (N _nominations_ =2846) based on other nomination (i.e. characteristics) that were given to these popular peers. Friends were also nominated as someone they liked (84.0%). Only 0.5% of the friends were also seen as unkind and/or mean, and a small group of friends was admired (10.8%).

**Figure S5**

*Distribution of direction of the presented social information across age and social source (within-variable).*

*Note.* The plots show the distribution of friend- (panel A) and popular peer trials (Panel B) across different ages (x-axis), with bars stacked by the type of social information that has been presented. Panel A and B show a similar division of type of social information within the friend and popular peer condition.

**Figure S6**
*Distribution of direction of peer choice (less or more cards) is equal across age and social condition (between-variable).*

*Note.* (A) displays an equal distribution of direction of peer choice (less or more cards) across age within the observation condition. (B) displays an equal distribution of direction of peer choice (less or more cards) across age within the private condition.

**Figure S7**

*Distribution of the SI distance from the participant’s initial choice (SI-C1)*

*Note.* The figure displays the difference in distance between the participant’s initial choice (C1) and the presented social information (SI; i.e. peer choice) for all trials. Peer choices that were presented to the participant within the experiment showed a similar distribution within the ‘more cards’ domain and ‘less cards’ domain.

**Figure S8**

*Distribution plot of the level of contamination within the friend and popular peer group*

*Note.* This figure illustrates the distribution of contamination scores for each peer group used in the experimental manipulation: the friend group and popular peer group. These contamination scores are calculated per participant and per social group. In the 'Friend group', scores were based on the proportion of friends that were also seen as popular (friends seen as poplar/ total number of friends). Conversely, in the 'Popular peer group', scores reflect the proportion of popular peers that were also seen as friend (popular peers seen as friend/ total number of popular peers). Each histogram provides insight into the frequency and variation of these scores across the sample, per peer group.

**Figure S9**

*Participant’s choice adjustment after observing their peer’s choice*

*Note.* A greater shift in the numbers of cards, in the direction of the social information (i.e. peer choice) occurred when participants observed more risky choices (highest panel) compared to when they observed more safe choices (lowest panel).

**Supplementary Tables**

**Table S1**

*Peer items and related questions that were asked during the peer nomination questionnaire*

| **Peer nomination items** | **Used in the study to define social sources** |
| --- | --- |
| Friend (Who are your friends?) | Yes |
| Best friend (Which classmate(s) is/are your best friends?) | Yes |
| Most popular (Which classmates are most popular in your class?) | Yes |
| Kind (Which classmates do you like?) | No |
| Not kind (Which classmates do you not like?) | No |
| Trustworthy (With whom would you share a secret or your feelings (e.g. that you are in love)) | No |
| Influential (Which classmates influence others to do what they want?) | No |
| Leader (Which classmates take often the lead in your group/class?) | No |
| Smart (which classmates are smart?) | No |
| Prosocial (Which classmates helps you with problems (e.g. homework, fixing your bike, cheer you up))? | No |
| Best friend (Which classmate(s) is/are your best friends?) | No |
| Looked up/ admired (Which classmate do you look up to?) | No |
| Cool (Which classmates are cool?) | No |
| Mean (Which classmates are mean? (e.g. a classmate who bullies excludes or humiliates someone) | No |
| Risky (Who does risky things?) | No |

**Table S2**

*Exclusion steps during data processing*

| **Order of data-filtering Exclusion criteria’s** | **Removal of data (N)** |
| --- | --- |
| 1. Participants who turn over zero cards in all trials or all possible cards in all trials. | 5 participants |
| 2. Participants that were unintentionally debriefed about the specific goal of the study due error in task-order | 3 participants |
| 3. Participant for whom it was not possible to implement both peer conditions correctly (e.g. their nominated friends and popular peers did not participate, thus social information from these groups was lacking) | 61 participants |
| 4. Participants who faced incorrect, and therefore, unrealistic social information due a programming error (incorrect linking between trial type and the belonging social information) | 7 participants |
| 5. Participants in the observation condition who were told to be observed by a peer that was eventually not present during session 2 (i.e. due illness) ^1^ | 41 participants |
| 6. Trials on which no social adjustment was possible (X-C1=0) ^2^ | 702 trials |
| 7. Trials that showed SI that is only 3 or less cards off the initial choice and trials that showed SI that is 20 cards or more off the initial choice will be deleted from the analysis ((absolute (X- C1) > 3 < 20) ^3^ | 842 trials with SI<3,  182 trials with SI>=20 |
| 8. Participants with less than 3 trials per peer condition after trial filtering ^4^ | 7 participants |
| 9. Participants with a mean S value (calculated per peer condition) which is 2 standard deviations from the group level mean S for that specific peer condition will be removed. | 7 participants |

*Note.* ^1^	Observing peers were chosen during the preparation phase of the social version of the game, for each participant. Due absence (i.e. illness) some observation peers were not present in class when participants played the social version of the task. ^2^ To increase believability, and if possible, one play round per social source condition was programmed as such that the social information (X) was in line with the initial choice of the participant. However, these trials are removed from the analysis as no adjustment toward the social information is possible. ^3^ Play rounds with less than 3 cards off the initial choice of the participant would represent social adjustment (if 0 <S< 1) from low impact, in which participants have only a small window for adjustment. Moreover, previous studies show that the social adjustment is dependent on how far the information is off your own choice. The selection of peer choices for use within the experiment was restricted based on the SI difference > 3 < 20 criteria; however, this restriction was not always feasible. ^4^ Participants with only 3 trials left for a peer condition were removed, as it was determined that estimating the participant’s adjustment score for this peer condition accurately would not be possible anymore.

**Table S3**
*Results of models predicting social adjustments (S) when correcting for variations in gender match between the social source of the experiment and the participant.*

|  | **Model 1** | **Model 2** | **Model 3** |
| --- | --- | --- | --- |
| Predictors | β (95 % CI)  p-value | β (95 % CI)  p-value | β (95 % CI)  p-value |
| (intercept) | 0.48 (0.38 – 0.58)  p<0.001 | 0.77 (0.68 – 0.87) p<0.001 | 0.79 (0.68 – 0.89) p<0.001 |
| Social source [popular] | -0.08 (-0.15 – -0.02) p=0.015 | -0.08 (-0.14 – -0.01)  p= 0.019 | -0.06 (-0.14 – 0.02) p=0.118 |
| Observation condition [yes] | -0.06 (-0.15 – 0.02) p=0.154 | -0.03 (-0.11 – 0.05) p= 0.471 | -0.03 (-0.11 – 0.05) p=0.438 |
| Social source [popular] ×  Observation condition [yes] | 0.08 (-0.01 – 0.17) p=0.072 | 0.07 (-0.01 – 0.16) p=0.098 | 0.08 (-0.01 – 0.17) p=0.080 |
| Direction [fewer cards] |  | -0.66 (-0.71 – -0.61) p<0.001 | -0.66 (-0.71 – -0.61) p<0.001 |
| Gender Match [same] | -0.03 (-0.12 – 0.05)  p=0.474 | -0.04 (-0.12 – 0.05)  p=0.392 | -0.04 (-0.12 – 0.04) p=0.352 |
| Age scaled (continuous) |  |  | -0.01 (-0.05 – 0.03) p=0.642 |
| Age scaled (Quadratic) |  |  | -0.01 (-0.05 – 0.04) p=0.754 |
| Age scaled (continuous) x  Social source [popular] |  |  | 0.02 (-0.03 – 0.06)  p=0.422 |
| Age scaled (Quadratic) x Social source [popular] |  |  | -0.02 (-0.06 – 0.03) p=0.510 |
| Number of participants  (N classes) | 457 (35) | 457 (35) | 457 (35) |
| Number of observations | 7548 | 7548 | 7548 |

*Note.* Linear mixed-effects models were fitted to choice adjustments, using ‘participant’ as a random intercept. As pre-registered, the gender match between the social source and the participant was also included to account for this potential covariate. However, note that the experiment was able to present a gender matched source for 87% of the participants. Including this variable, coding gender (non)matches, did not affect the main results of the model. The estimated effects are presented with 95% confidence intervals (CIs) in parentheses and P values below the CIs.

**Table S4**
*Results of models predicting social adjustments (S) when correcting for any impurities (contaminations) in the two social source conditions.*

|  | **Model 1** | **Model 2** | **Model 3** |
| --- | --- | --- | --- |
| Predictors | β (95 % CI)  p-value | β (95 % CI)  p-value | β (95 % CI)  p-value |
| (intercept) | 0.44 (0.37 – 0.50)  p<0.001 | 0.73 (0.67 – 0.79) p<0.001 | 0.74 (0.68 – 0.81) p<0.001 |
| Social source [popular] | -0.09 (-0.16 – -0.01) p=0.009 | -0.08 (-0.14 – -0.02)  p= 0.015 | -0.06 (-0.14 – 0.01) p=0.106 |
| Observation condition [yes] | -0.06 (-0.15 – 0.02) p=0.146 | -0.03 (-0.11 – 0.05) p= 0.454 | -0.03 (-0.11 – 0.05) p=0.429 |
| Social source [popular] ×  Observation condition [yes] | 0.08 (-0.01 – 0.17) p=0.074 | 0.07 (-0.01 – 0.16) p=0.101 | 0.08 (-0.01 – 0.17)  p= 0.085 |
| Contamination Score | 0.08 (-0.02 – 0.19) p = 0.120 | 0.07 (-0.03 – 0.17)  p= 0.157 | 0.07 (-0.03 – 0.17)  p= 0.149 |
| Direction [fewer cards] |  | -0.65 (-0.70 – -0.60) p<0.001 | -0.65 (-0.70 – -0.60) p<0.001 |
| Age scaled (continuous) |  |  | -0.01 (-0.05 – 0.03)  p= 0.715 |
| Age scaled (Quadratic) |  |  | -0.01 (-0.05 – 0.03)  p= 0.715 |
| Age scaled (continuous) x  Social source [popular] |  |  | 0.02 (-0.03 – 0.06)  p= 0.455 |
| Age scaled (Quadratic) x Social source [popular] |  |  | -0.02 (-0.06 – 0.03) p= 0.497 |
| Number of participants  (N classes) | 457 (35) | 457 (35) | 457 (35) |
| Number of observations | 7562 | 7562 | 7562 |

*Note.* Linear mixed-effects models were fitted to choice adjustments, using ‘participant’ as a random intercept. Model 1 tested whether participants’ choice adjustments were influenced by the social source (popular peer versus friend) and whether this effect was dependent on the social condition (observation versus private). Model 2 included the direction of the peer choice (fewer cards versus more cards) as a covariate to test whether participants’ choice adjustments were influenced by direction. Exploratory age effects were tested in Model 3. The estimated effects are presented with 95% confidence intervals (CIs) in parentheses and P values below the CIs.

**Table S5**
*Results of sensitivity model predicting social adjustments (S) when excluding (N=150) participants with a contamination above 40% (contamination score > 0.4) for one of the social source conditions.*

|  | **Model 1** | **Model 2** | **Model 3** |
| --- | --- | --- | --- |
| Predictors | β (95 % CI)  p-value | β (95 % CI)  p-value | β (95 % CI)  p-value |
| (intercept) | 0.44 (0.36 – 0.50)  p<0.001 | 0.73 (0.67 – 0.80) p<0.001 | 0.74 (0.65 – 0.83) p<0.001 |
| Social source [popular] | -0.09 (-0.17 – -0.01) p=0.023 | -0.08 (-0.164 – -0.00)  p= 0.038 | -0.08 (-0.18 – 0.01) p=0.086 |
| Observation condition [yes] | -0.08 (-0.19 – 0.02) p=0.128 | -0.05 (-0.15 – 0.05) p= 0.372 | -0.05 (-0.15 – 0.05) p=0.367 |
| Social source [popular] ×  Observation condition  [yes] | 0.10 (-0.01 – 0.21) p=0.080 | 0.09 (-0.02 – 0.20) p=0.093 | 0.09 (-0.02 – 0.20)  p= 0.105 |
| Contamination Score | 0.06 (-0.40 – 0.53 p = 0.790 | 0.08 (-0.35 – 0.50)  p= 0.717 | 0.07 (-0.35 – 0.50)  p= 0.731 |
| Direction [fewer cards] |  | -0.65 (-0.71 – -0.59) p<0.001 | -0.65 (-0.72 – -0.59) p<0.001 |
| Age scaled (continuous) |  |  | -0.00 (-0.05 – 0.05)  p= 0.900 |
| Age scaled (Quadratic) |  |  | -0.01 (-0.06 – 0.04)  p= 0.650 |
| Age scaled (continuous) x  Social source [popular] |  |  | -0.01 (-0.06 – 0.05)  p= 0.743 |
| Age scaled (Quadratic) x Social source [popular] |  |  | 0.00 (-0.05 – 0.06) p= 0.939 |
| Number of participants  (N classes) | 307 (33) | 307 (33) | 307 (33) |
| Number of observations | 5023 | 5023 | 5023 |

*Note.* Linear mixed-effects models were fitted to choice adjustments, using ‘participant’ as a random intercept. Model 1 tested whether participants’ choice adjustments were influenced by the social source (popular peer versus friend) and whether this effect was dependent on the social condition (observation versus private). Model 2 included the direction of the peer choice (fewer cards versus more cards) as a covariate to test whether participants’ choice adjustments were influenced by direction. Exploratory age effects were tested in Model 3. The estimated effects are presented with 95% confidence intervals (CIs) in parentheses and P values below the CIs.

**Table S6**

*Participation rate per class*

| **Classcode** | **150** | **151** | **152** | **153** | **154** | **155** | **156** | **157** | **160** | **162** | **163** | **164** | **165** | **166** | **168** | **173** |
| --- | --- | --- | --- | --- | --- | --- | --- | --- | --- | --- | --- | --- | --- | --- | --- | --- |
| **Participation rate (%)** | 90 | 86.2 | 65.2 | 91.7 | 83.3 | 77.8 | 100 | 84.2 | 100 | 64.3 | 69 | 95.8 | 69.2 | 100 | 100 | 100 |
| **Classcode** | **175** | **176** | **181** | **182** | **183** | **184** | **185** | **186** | **187** | **188** | **189** | **190** | **192** | **193** | **194** | **199** |
| **Participation rate (%)** | 70.6 | 100 | 92.9 | 100 | 96.2 | 72 | 81 | 66.7 | 89.7 | 87.5 | 87.5 | 69.2 | 73.1 | 70.6 | 96.4 | 96.6 |

*Note.* The participation rate mainly reflects the number of parents who did give consent for their children to participate in the study. Additionally, this participation rate was affected by adolescents who were absent during session 1 due to reasons such as illness. Due to absence during session 1, these pupils could also not participate in session 2. Among all participating classes, 12 students themselves decided not to take part in the study. The reason for this is unknown.

**Supplementary Methods**

**Preparation of the social information**

The choices that were shown during the experimental task, were real choices pooled from a group of friends or a group of popular peers. Based on the peer nomination questionnaire participating (best) friends were chosen as source. For the popular peer condition information stemmed from classmates that were nominated by the participant as being ‘most popular’ + classmates who received the top 5 highest nomination score on this item on class level (sum score of all nominations given by the classroom to each classmate).

The peer choice that was selected to be shown during the task was selected based on the following criteria:
1) The choice needs to be between 5 cards-off and 7 cards-off, if not possible

2) between 5 cards-off and 11 cards-off, if not possible

3) between 5 cards-off and 19 cards-off, if not possible

4) 3 cards-off or 4 cards-off, if not possible

4) less than 2 cards-off, if not possible

5) get the maximum SI distance that is available.

If possible, one trial per peer condition was included that showed social information that was 0 or 1 card off the participants’ own choice, to increase believability of the social information.

Efforts were made to minimalize the difference (i.e. direction of the choice and distance between the choice of the peer and the participant’s previous choice) in peer choices presented in the friend and popular condition. However, as the experiment used real data, this was only controllable to some extent.

In the friend condition aimed to present a best-friend name in the instruction (and as observation peer in the observation condition). If not possible, a (same gender) friend was selected. The popular condition aimed to present a popular peer name in the instruction (and as observation peer in the observation condition), based on the following criteria: The popular peer belongs to the top 5 peers with the highest popularity nominations on class level. If possible, a gender-matched popular peer is selected. It was verified whether the popular peer was also nominated as popular by the participant. Preference was given to popular peers that were also seen as popular by the participant (and thus not only by their classmates).

**Allocation procedure**

Participants within one class were randomly assigned to either the observation or the private condition. However, if most of the pre-selected peers for the observation condition turned out to be absent (thus peers who were selected to be the peer with whom choices would be shared), the whole class received the private condition, to limit the loss of data (see section ‘exclusion’). In response to this, to balance the sample over the two peer conditions and to obtain a balanced age group within both conditions, it also happened that all participants of one class were assigned to the observation condition if all observational peers were present in class.
